# Neuronal Nitric Oxide Synthase Contributes to PTZ Kindling-Induced Cognitive Impairment and Depressive-Like Behavior

**DOI:** 10.3389/fnbeh.2017.00203

**Published:** 2017-10-18

**Authors:** Xinjian Zhu, Jingde Dong, Bing Han, Rongrong Huang, Aifeng Zhang, Zhengrong Xia, Huanhuan Chang, Jie Chao, Honghong Yao

**Affiliations:** ^1^Department of Pharmacology, Medical School of Southeast University, Nanjing, China; ^2^Department of Geriatric Neurology, Nanjing Brain Hospital Affiliated to Nanjing Medical University, Nanjing, China; ^3^Department of Pathology, Medical School of Southeast University, Nanjing, China; ^4^Analysis and Test Center of Nanjing Medical University, Nanjing, China; ^5^Nanjing Biomedical Research Institute of Nanjing University, Nanjing, China; ^6^Department of Physiology, Medical School of Southeast University, Nanjing, China

**Keywords:** epilepsy, neuronal nitric oxide synthase, cognitive impairment, depressive-like behavior

## Abstract

Epilepsy is a chronic neurological disease which is usually associated with psychiatric comorbidities. Depsression and cognition impairment are considered to be the most common psychiatric comorbidities in epilepsy patients. However, the specific contribution of epilepsy made to these psychiatric comorbidities remains largely unknown. Here we use pentylenetetrazole (PTZ) kindling, a chronic epilepsy model, to identify neuronal nitric oxide synthase (nNOS) as a signaling molecule triggering PTZ kindling-induced cognitive impairment and depressive-like behavior. Furthermore, we identified that both hippocampal MAPK and PI3K/AKT signaling pathways were activated in response to PTZ kindling, and the increased MAPK and PI3K/AKT signaling activation was paralleled by increased level of reactive oxygen species (ROS) in the hippocampus. However, the PTZ kindling-induced MAPK, PI3K/AKT signaling activities and the ROS level were attenuated by nNOS gene deficiency, suggesting that nNOS may act through ROS-mediated MAPK and PI3K/AKT signaling pathways to trigger cognition deficit and depressive-like behavior in PTZ-kindled mice. Our findings thus define a specific mechanism for chronic epilepsy-induced cognitive impairment and depressive-like behavior, and identify a potential therapeutic target for psychiatric comorbidities in chronic epilepsy patients.

## Introduction

Psychiatric comorbidities are relatively common in epilepsy patients. Among these comorbidities, depression and cognition impairment appear to be the major comorbidities associated with chronic epilepsy (Gaitatzis et al., 2004; Kanner, 2006, 2016; LaFrance et al., 2008; Loughman et al., 2016; Tai et al., 2016). Despite accumulating epidemiological and animal model evidence suggesting a correlation between epilepsy and the psychiatric comorbidities of depression and cognitive deficit, the biological mechanisms underlying this correlation remains poorly understood. Neuronal nitric oxide synthase (nNOS) has been widely distributed in the neurons, where it produces nitric oxide (NO) in the process of converting L-arginine into citrulline, with the presence of NADPH. NO in the brain has been involved in synaptogenesis, neural transmission, learning and memory and synaptic plasticity. nNOS has been largely distributed in the brain regions of striatum, hippocampus, hypothalamus and amygdala, where it is involved in the regulation of cognition and affective behaviors (Downen et al., 1999; Kittner et al., 2003; Saavedra et al., 2013). Indeed, increasing evidence has shown that nNOS plays a pivotal role in multiple psychiatry disorders, including schizophrenia, depression and anxiety (Kittner et al., 2003; Reif et al., 2006; Brzustowicz, 2008; Delorme et al., 2010; Lawford et al., 2013; Saavedra et al., 2013). MAPK and PI3K/AKT are two major intracellular signaling pathways involved in the brain activities. Recent studies demonstrate that MAPK (Hutton et al., 2017; Ullrich et al., 2017) and PI3K/AKT (Papaleo et al., 2016) signaling pathways are associated with a number of neuropsychiatric disorders. However, whether these signaling pathways are dependent on nNOS activities remains unclear.

Pentylenetetrazole (PTZ) kindling is a chronic epilepsy model, in which a progressive seizure development is observed. PTZ kindling causes alterations in the molecular and cellular levels, which are responsible for neuronal plasticity. It has been demonstrated that PTZ kindling-induced morphological changes are usually accompanied with long-lasting changes in emotional behavior (Franke and Kittner, 2001; Mortazavi et al., 2005). A recent study reports that PTZ kindling induces depression-like behavior and cognition deficits (Choudhary et al., 2013), suggesting chronic epilepsy is associated with psychiatric comorbidities.

In our previous study, we demonstrated that hippocampal nNOS expression and enzymatic activity have been dramatically enhanced after the mice were kindled (Zhu et al., 2016a). Here, in this study, we hypothesize that this increased nNOS activity acts through MAPK and PI3K/AKT signaling pathways to trigger cognition deficit and depressive-like behavior in PTZ-kindled mice.

## Materials and methods

### Animals

Male C57BL/6J mice (4–6 weeks old; weighing 19 ± 2 g at the beginning of the experiments) were obtained from Nanjing Biomedical Research Institute of Nanjing University (NBRI) (Nanjing, China). Mice lacking nNOS (B6;129S4-Nos1^tm1Plh^) obtained from NBRI were backcrossed to C57BL/6J strain and the heterozygotes were intercrossed to obtain mutation homozygotes. Male homozygous nNOS-null (nNOS^−/−^) and their wild-type (nNOS^+/+^) littermates (4–6 weeks old) were used in the experiment. The animals were housed in plastic cages and kept in a regulated environment (22 ± 1°C) with an artificial 12 h light/dark cycle (lighted from 7:00 a.m. to 7:00 p.m.). Food and tap water were available *ad libitum*. Procedures for PTZ induced-kindling and all subsequent experiments were approved by the Animal Care and Use Committee of Medical School of Southeast University. All efforts were made to minimize animal suffering and discomfort and to reduce the number of animals used.

### PTZ kindling procedure

**Figure 1:**
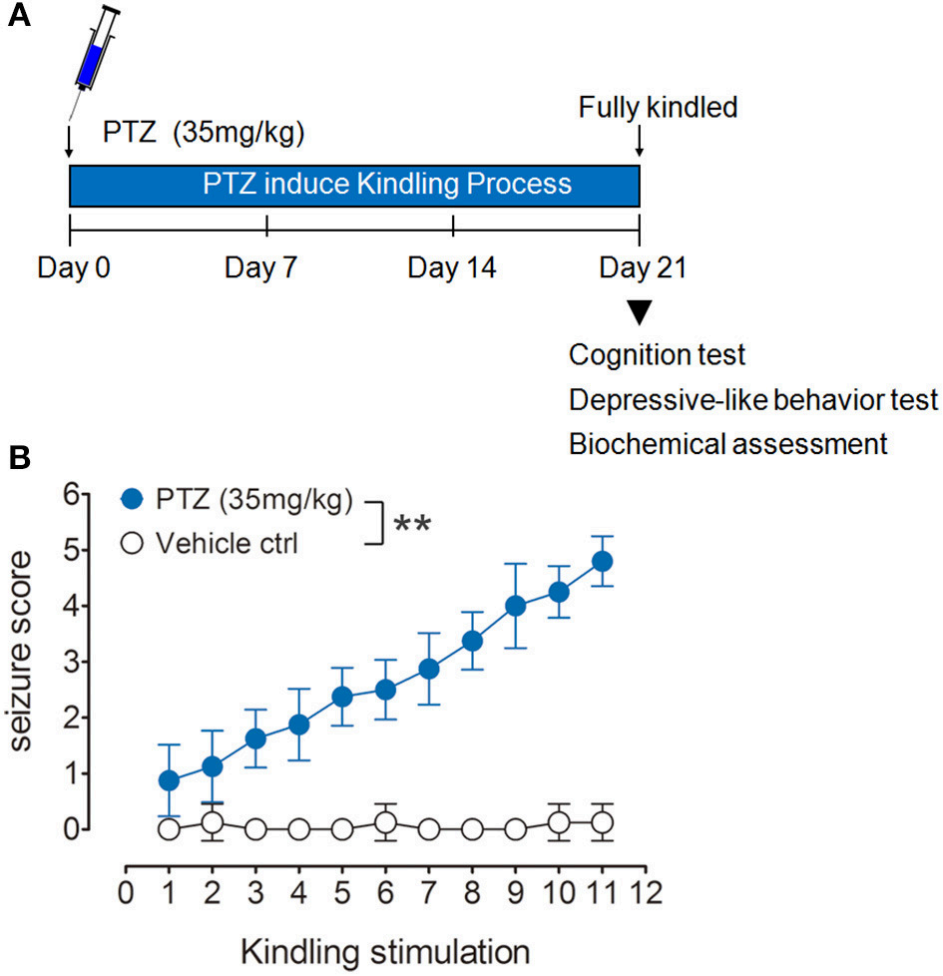
PTZ-induced kindling model. **(A)** Schematic representation of experimental design. Mice were repeatedly treated with 35 mg/kg PTZ every other day to induce kindling, immediately after these mice were fully kindled, they were subject to cognition test, depressive-like behavior test and biochemical assessment. **(B)** Kindling was evoked by repeatedly and intermittently treating mice with PTZ at a dose of 35 mg/kg once every other day for 11 total injections. The mice showing more than three consecutive stage 4 seizures were considered to be fully kindled (*n* = 8). Values are means ± S.E.M. ^**^p < 0.01 compared with vehicle control mice, repeated measures ANOVA.

PTZ kindling model was produced as we previously described (Zhu et al., 2015). Briefly, mice were treated with a subconvulsive dose of PTZ (Sigma Aldrich, St. Louis, MO, USA) at 35 mg/kg intraperitoneally on every second day for eleven total injections. (Figure 1A). Vehicle control mice received the same amount of saline. After each PTZ injection, convulsive behaviors were observed for 30 min by a video monitoring system (HK vision, Hanzhou, China). The seizure intensity was evaluated by using the following scale. Stage 0, no response; Stage 1, ear and facial twitching; Stage 2, convulsive twitching axially through the body; Stage 3, myoclonic jerks and rearing; Stage 4, turning over onto the side, wild running, and wild jumping; Stage 5, generalized tonic-clonic seizures; and Stage 6, death (Schroder et al., 1993; Becker et al., 1995; Mizoguchi et al., 2011).

### Cognitive function evaluation

**Figure 2:**
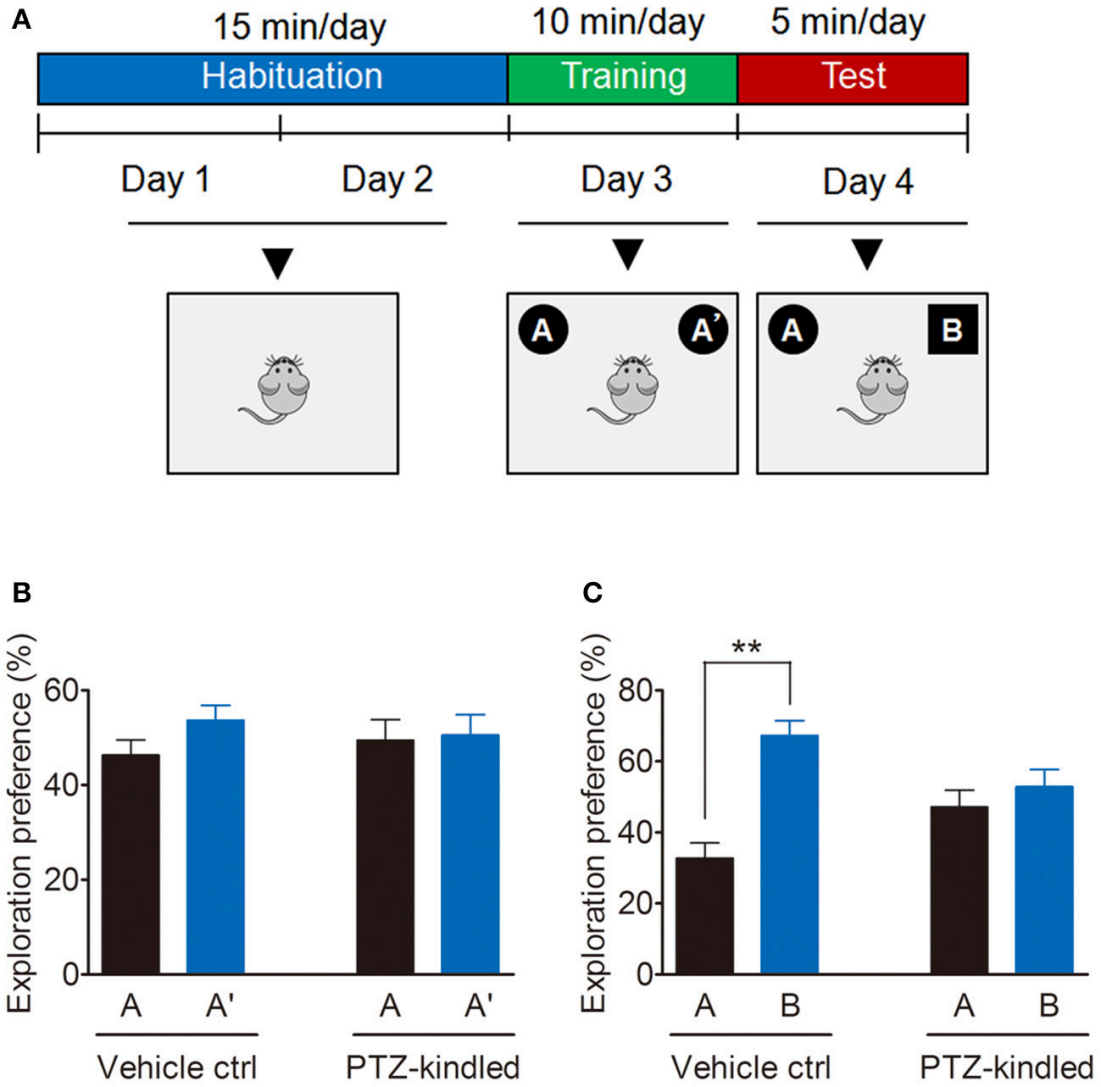
PTZ kindling-induced cognitive impairment. **(A)** Schematic of NOR test. Mice were habituated to the NOR arena in the absence of objects for 15 min per day for 2 consecutive days, after which they were exposed in the same arena for 10 min to two identical objects A and A' for training. 24 h later, they underwent a 5 min test in which one of the objects A' was replaced with a novel object B. **(B,C)** Bar graphs showing the exploration preference for the familiar and novel object (percent of time exploring each object) in vehicle control and PTZ-kindled mice (*n* = 8). Values are means ± S.E.M. ^**^p < 0.01, unpaired two-tailed Student's *t*-test.

Cognitive function evaluation is conducted by novel object recognition (NOR) test, which is based on a rodent's nature to preferentially investigate unfamiliar objects rather than familiar objects. NOR test was performed as previously described (Bevins and Besheer, 2006; Nomoto et al., 2016) with some modifications. Briefly, NOR testing is consisting of three phases: habituation, training and test (Figure 2A). During the habituation session, mice were placed in the empty arena and allowed to freely explore without objects 15 min per day for two consecutive days. During training session, mice were placed in the arena with two identical objects A and A', and were allowed to explore the objects for 10 min. During the test session, mice were placed in the arena, where one familiar object A' was replaced with a novel object B, and mice were allowed to explore the object for 5 min. The arena and the objects were cleaned thoroughly with 75% ethanol between trials to remove any olfactory cues. A video monitoring system (Hikvision video monitoring system, Hangzhou, China) was used to capture the animal behavior for later analysis. Time spent exploring each object was calculated by an observer blind to the experimental conditions and was expressed as a percentage of the total exploration time.

### Sucrose preference test

After 4 days of NOR test, the mice were recovered for 24 h and then subjected to 2 days of sucrose preference test. The sucrose preference test was used to assess the taste preference of sweetened water. A diminished preference for the sweetened water is a sign of anhedonia, indicating depressive-like behavior. The sucrose preference test was conducted as previously described (Strekalova et al., 2004; Snyder et al., 2011; Wu et al., 2014) with some modifications. Briefly, mice were given a free choice between two bottles, one with 1% sucrose solution and another with tap water for 48 h. To avoid place preference in drinking behavior, the position of the bottles was switched after 24 h. The consumption of tap water and sucrose solution was estimated simultaneously in control and experimental groups by weighing the bottles. The preference for sucrose was calculated as a percentage of consumed sucrose solution of the total amount of liquid drunk. The food preference is also calculated as a control to demonstrate that mice do not show a place preference.

### Tail-suspension test and forced swim test

After the sucrose preference test, the mice were recovered for 24 h and then subjected to tail-suspension test followed by forced swim test. The tail-suspension test was used to evaluate depressive-like behaviors in animals. The method is based on the observations that mice suspended by the tails show immobility, which reflects despair of hope. The tail-suspension test was performed as previously described (Steru et al., 1985) with small modifications. Each mouse was suspended at a height of 50 cm using a thread tied with the tip of tail. The mice were considered to be immobile when they did not show any movement of body and hanged passively. The duration of immobility was recorded throughout the 5 min test period. The forced swim test was created as a situation of despair and allows to assessing the depressive-like behavior. Although some studies indicate that FST reflects a passive stress coping and adaption mechanism (de Kloet and Molendijk, 2016; Commons et al., 2017), it is still the most widely used tool to test depressive-like behavior. The forced swim test was performed as previously described (Porsolt et al., 1977). Briefly, the mice were recovered for 24 h after the tail-suspension test and were placed in a transparent glass cylinder (height, 30 cm; diameter, 10 cm) filled with 20 cm height of water at 23 ± 1°C. Mice were judged to be immobile when if floated in an upright position and made only small movements to keep its head above water for more than 2 s. The duration of immobility during the 6 min of trial was recorded using the video monitoring system (Hikvision video monitoring system, Hangzhou, China). After each test, the container was washed and refilled with fresh water.

### Measurement of ROS production

ROS production was measured by a cell membrane-permeable superoxide-sensitive fluorescent dye dihydroethidium (DHE) (Sigma-Aldrich, St. Louis, MO, USA) as we previously described (Zhu et al., 2016b). Briefly, hippocampal sections were incubated in 1 μM DHE (in 0.1 M PBS, PH = 7.4) for 15 min in the dark room. Hippocampal sections were then rinsed with PBS three times and mounted on gelatin-coated slides. DHE fluorescence was detected by a confocal laser scanning microscope (Olympus LSM-GB200, Japan) using an excitation wavelength of 520–540 nm. Fluorescence was quantified with the Image J software program (NIH, Bethesda, MD, USA).

### Western blotting

Hippocampal tissues were lysed for 15 min in tissue lysis buffer (Beyotime Biotechnology, China). The protein concentration was measured using a BCA protein assay kit (Pierce, Rockford, IL, USA). Hippocampal proteins were then separated by 12% acrylamide denaturing gels (SDS-PAGE) and were transferred to nitrocellulose membranes (Amersham, Little Chalfont, UK) by a Bio-Rad mini-protein-III wet transfer unit (Hercules, CA, USA) overnight at 4°C.

The membranes were incubated with 5% non-fat milk in TBST (10 mmol/l Tris pH = 7.6, 150 mmol/L NaCl, 0.01%Tween-20) for 1 h at room temperature followed by several washes, then were incubated with rabbit anti-ERK and phospho-ERK (1:2,500; Abcam, Temecula, CA, USA), rabbit anti-p38 and phospho-p38 (1:2,000; Cell signaling, Danvers, MA, USA), mouse anti-PI3K (1:2,000; Cell signaling, Danvers, MA, USA), rabbit anti-AKT and phosphor-AKT (1:2,000; Cell signaling, Danvers, MA, USA) and rabbit anti-β-actin (1:5,000; Sigma-Aldrich, St, Louis, USA) in TBST overnight at 4°C. After several washes, the membranes were incubated with HRP-linked secondary antibody (Boster Bioengineering, Wuhan, China) diluted 1:5,000 for 1 h. After several washes, the antibody was detected by an enhanced chemiluminescence (ECL) (Millipore, Billerica, MA, USA) by using a MicroChemi chemiluminescent image analysis system (DNR Bio-imaging Systems, Jerusalem, Israel). Blots were quantified using the Image J software (NIH, Bethesda, MD, USA).

### Statistical analysis

All data are presented as the means ± S.E.M. Statistical significance was determined by using unpaired two-tailed Student's *t*-test for two group's comparison and by using two-way ANOVA for multi-group comparisons, and repeated-measures ANOVA. Tukey's test was used for *post-hoc* comparisons. A Spearman rank correlation coefficient was used to determine any correlation between the immobility duration under the condition of forced swim and tail suspension and the seizure score during kindling development. Differences were considered to be significant for values of *p* < 0.05.

## Results

### PTZ kindling induced cognitive impairment

The PTZ kindling model was successfully established by giving the mice with PTZ at a dose of 35 mg/kg every other day for 11 doses (Figure 1A). PTZ-kindled mice showed a gradual increase of seizure intensity, compared with the mice in control group (Figure 1B). PTZ kindling as a chronic epilepsy experimental model is usually associated with neuronal plasticity and causing psychiatric comorbidities. To determine whether PTZ kindling affects cognition and depressive-like behavior in mice, we performed a variety of neurobehavior tests 24 h after the mice were fully kindled. Firstly, we examined the cognitive function of PTZ-kindled mice by using a novel object recognition (NOR) test (Figure 2A). During the training phase, both PTZ-kindled and vehicle control mice spent almost the same percent of time exploring the two identical objects A and A' (Figure 2B). However, in the testing phase, the vehicle control mice spent more time exploring the novel object B compared to the familiar object A (Figure 2C), indicating that they remembered the familiar object A from the training phase and thus had a preference for the novel object B in the testing phase. In contrast, during the testing phase, PTZ-kindled mice spent almost the same amount of time exploring both the novel object B and the familiar object A (Figure 2C), suggesting that these mice did not remember the familiar object A during the training phase and had a cognitive deficit.

### PTZ kindling induced depressive-like behavior

**Figure 3:**
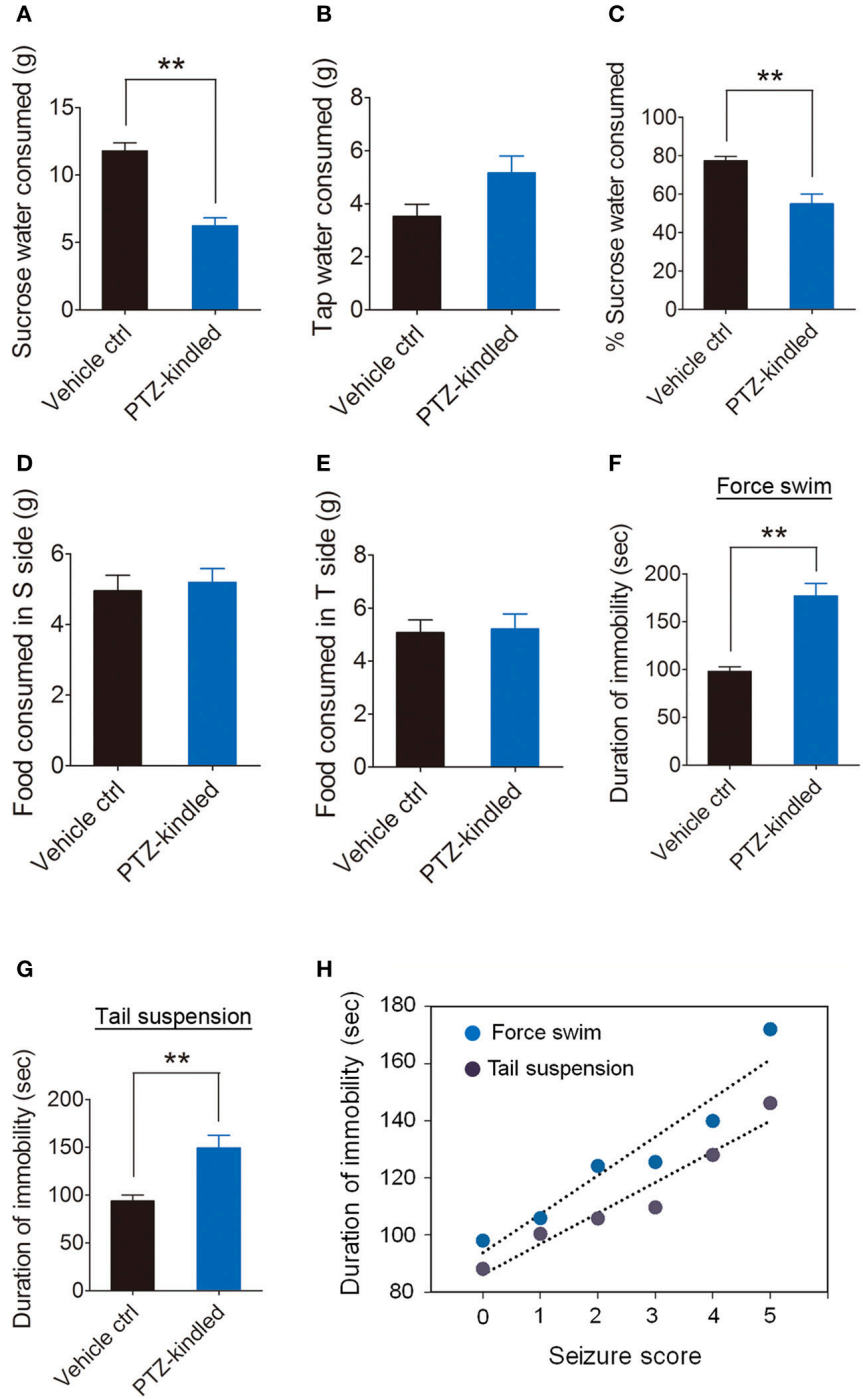
PTZ kindling-induced depressive-like behavior. **(A–E)** Bar graphs showing the sucrose water consumed, tap water consumed, percent of sucrose water consumed, food consumed in sucrose (S) side and food consumed in tap water (T) side in vehicle control and PTZ-kindled mice in assessment of depressive-like behavior using the sucrose preference (SP) tests (*n* = 8). **(F–G)** Bar graphs showing the immobility time in vehicle control and PTZ-kindled mice in assessment of depressive-like behavior using force swim and tail suspension test (*n* = 8). **(H)** Seizure scores of animals during PTZ-kindling development are plotted against immobility time in force swim (*p* = 0.002, *r* = 0.94) and tail suspension test (*p* = 0.003, *r* = 0.96) (*n* = 8). Values are means ± S.E.M. ^**^p < 0.01, unpaired two-tailed Student's *t*-test. The coefficient of correlation (*r*) is calculated using the Spearman test.

We examined the depressive-like behaviors by using sucrose preference, forced swim and tail suspension test. Our data show that PTZ-kindled mice showed significantly reduced sucrose water consumption (Figure 3A) and the percentage of sucrose water consumption (Figure 3C) compared to vehicle control mice. However, the tap water (Figure 3B) and food consumption (Figure 3D,E) between these two group of mice remains similar. Immobility in forced swim and tail-suspension test represents a symptom of depression. Our data show that PTZ-kindled mice in both forced swim (Figure 3F) and tail-suspension test (Figure 3G) displayed significant increase of immobility duration, suggesting these mice had depressive-like behavior. Furthermore, we examined the immobility duration under the conditions of the forced swim and tail suspension correlated with the severity of behavioral seizures during kindling process in individual mice. Our data show a strong positive correlation between the duration of immobility and the seizure score during PTZ kindling development in forced swim as well as tail-suspension test (Figure 3H). Taken together, these results suggest that PTZ kindling induced cognitive impairment and depressive-like behaviors.

### PTZ kindling-induced cognitive impairment and depressive-like behavior is dependent on nNOS activity

**Figure 4:**
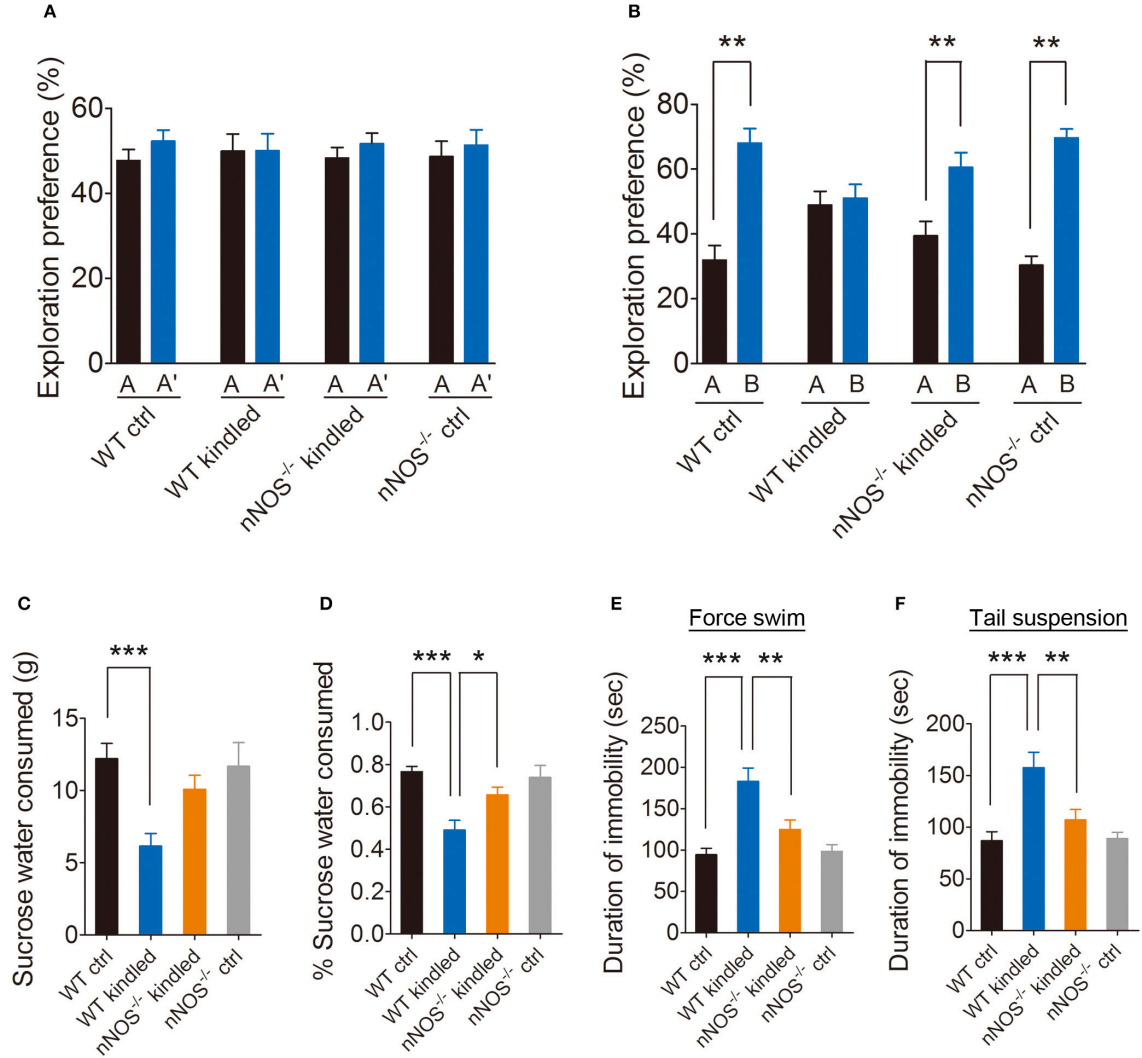
PTZ kindling-induced cognition deficit and depressive-like behavior is dependent on nNOS activity. **(A,B)** Bar graphs showing the exploration preference for the familiar and novel object (percent of time exploring each object) in WT ctrl, WT kindled, nNOS^−/−^ ctrl and nNOS^−/−^ kindled mice (*n* = 8). Values are means ± S.E.M. ^**^p < 0.01, unpaired two-tailed Student's *t*-test. **(C,D)** Bar graphs showing the sucrose water consumption and percent of sucrose water consumed in WT ctrl, WT kindled, nNOS^−/−^ ctrl and nNOS^−/−^ kindled mice in assessment of depressive-like behavior using the SP test (*n* = 8). **(E,F)** Bar graphs showing the immobility time in WT ctrl, WT kindled, nNOS^−/−^ ctrl and nNOS^−/−^ kindled mice in assessment of depressive-like behavior using force swim and tail suspension test (*n* = 8). Values are means ± S.E.M. ^*^p < 0.05, ^**^p < 0.01, ^***^p < 0.001.

Growing body of evidence demonstrated that nNOS plays a pivotal role in psychiatry disorders, to confirm the involvement of nNOS in PTZ kindling-induced cognitive impairment and depressive-like behavior, we tested the cognition and depressive-like behavior in nNOS^−/−^ mice and their wildtype littermates under normal and PTZ kindling conditions. Firstly, we examined the cognitive function by using NOR test as described above (Figure 2A). During the training phase, the wildtype control, wildtype kindled, nNOS^−/−^ control and nNOS^−/−^ kindled mice spent almost the same percent of time exploring the two identical objects A and A' (Figure 4A). However, in the testing phase, the vehicle control mice, nNOS^−/−^ control and nNOS^−/−^ kindled mice spent more time exploring the novel object B compared to the familiar object A (Figure 4B), indicating that these mice remembered the familiar object A from the training phase and thus had a preference for the novel object B in the testing phase. In contrast, during the testing phase, the wildtype kindled mice spent almost the same amount of time exploring both the novel object B and the familiar object A (Figure 4B), indicating that these mice did not remember the familiar object A during the training phase and had a cognitive deficit. These data suggests that PTZ kindling induced cognitive impairment. Depletion of nNOS rescued PTZ kindling-induced cognitive deficit.

We then examined the depressive-like behaviors by using sucrose preference, forced swim and tail suspension test. Our data show that wildtype kindled mice showed significantly reduced percentage of sucrose water consumption compared to wildtype control mice (Figure 4D), moreover, nNOS^−/−^ kindled mice showed increased percentage of sucrose water consumption compared to wildtype kindled mice (Figure 4D). nNOS^−/−^ control and wildtype control mice exhibited similar percentage of sucrose water consumption (Figure 4D). For sucrose water consumption, two-way ANOVA revealed a significant main effect of PTZ treatment [*F*_(1, 28)_ = 14.69, *p* < 0.001], but not a significant effect of genotype [*F*_(1, 28)_ = 1.79, *p* = 0.191], nor a significant effect of PTZ treatment × genotype interaction [*F*_(1, 28)_ = 2.18, *p* = 0.15]. For the percentage of sucrose water consumption, two-way ANOVA revealed a significant main effect of PTZ treatment [*F*_(1, 28)_ = 17.63, *p* < 0.001], but not a significant effect of genotype [*F*_(1, 28)_ = 2.30, *p* = 0.14], however, there is a significant effect of PTZ treatment × genotype interaction [*F*_(1, 28)_ = 5.11, *p* = 0.032]. A Tukey *post-hoc* test revealed that WT kindled mice showed a significant lower percentage of sucrose water consumption than WT ctrl mice (*p* < 0.001), and nNOS^−/−^ kindled mice showed a significant higher percentage of sucrose water consumption than WT kindled mice (*p* = 0.015). In forced swim and tail-suspension test, we found that wildtype kindled mice displayed significant increase of immobility duration compared to wildtype control mice in both forced swim (Figure 4E) and tail-suspension test (Figure 4F). Furthermore, nNOS^−/−^ kindled mice displayed significant decrease of immobility duration compared to wildtype kindled mice in both of these two tests (Figures 4E,F), while nNOS^−/−^ control and wildtype control mice have similar immobility duration in both of these two test (Figures 4E,F). For the immobility time in force swim test, two-way ANOVA revealed a significant main effect of PTZ treatment [*F*_(1, 28)_ = 25.06, *p* < 0.001], genotype [*F*_(1, 28)_ = 4.94, *p* = 0.034], as well as PTZ treatment × genotype interaction [*F*_(1, 28)_ = 4.74, *p* = 0.011]. A Tukey *post-hoc* test revealed that WT kindled mice had longer duration of immobility than WT ctrl mice (*p* < 0.001), and nNOS^−/−^ kindled mice had shorter duration of immobility than WT kindled mice (*p* = 0.002). For the immobility time in tail suspension test, two-way ANOVA revealed a significant main effect of PTZ treatment [*F*_(1, 28)_ = 24.36 *p* < 0.001], genotype [*F*_(1, 28)_ = 5.02, *p* = 0.033], as well as PTZ treatment × genotype interaction [*F*_(1, 28)_ = 4.40, *p* = 0.045]. A Tukey *post-hoc* test revealed that WT kindled mice had longer duration of immobility than WT ctrl mice (*p* < 0.001), and nNOS^−/−^ kindled mice had shorter duration of immobility than WT kindled mice (*p* = 0.006). These data suggests that PTZ kindling induced depressive-like behavior. Depletion of nNOS suppressed PTZ kindling-induced depressive-like behavior. Taken together, these results indicated that PTZ kindling-induced cognitive impairment and depressive-like behavior is dependent on nNOS activity.

### PTZ kindling-induced hippocampal ROS production is dependent on nNOS activity

**Figure 5:**
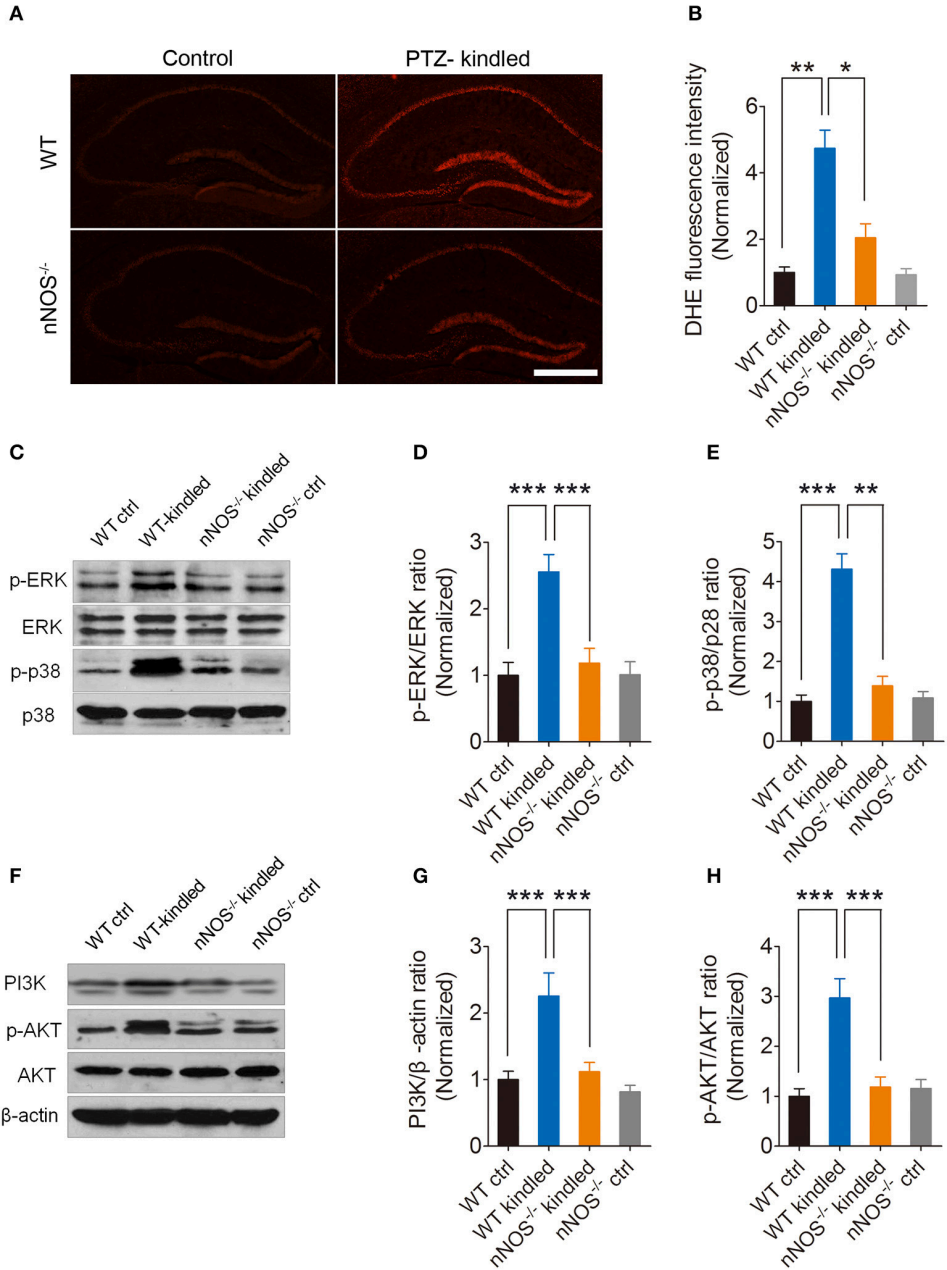
PTZ kindling induces nNOS-dependent ROS production and activates nNOS-dependent MAPK and PI3K/AKT signaling pathways. **(A)** Representative images of DHE fluorescence in the hippocampus of WT ctrl, WT kindled, nNOS^−/−^ ctrl and nNOS^−/−^ kindled mice. **(B)** Bar graph showing the quantification of the DHE fluorescence intensity, which represents the ROS levels in the hippocampus of WT ctrl, WT kindled, nNOS^−−/−−^ ctrl and nNOS^−/−^ kindled mice (*n* = 5). **(C)** Western blots showing the protein levels of p-ERK, ERK p-p38 and p38 in WT ctrl, WT kindled, nNOS^−/−^ ctrl and nNOS^−/−^ kindled mice. **(D,E)** Bar graphs showing the quantification of ERK and p38 phosphorylation levels which were represented as the ration of p-ERK/ERK and p-p38/p38 in WT ctrl, WT kindled, nNOS^−/−^ ctrl and nNOS^−/−^ kindled mice (*n* = 5). **(F)** Western blots showing the protein levels of PI3K, p-AKT and AKT in WT ctrl, WT kindled, nNOS^−/−^ ctrl and nNOS^−/−^ kindled mice. **(G,H)** Bar graphs showing the quantification of PI3K and AKT phosphorylation level which were represented as the ratio of PI3K/β-actin and p-AKT/AKT in WT ctrl, WT kindled, nNOS^−/−^ ctrl and nNOS^−/−^ kindled mice (*n* = 5). Values are means ± S.E.M. ^*^p < 0.05, ^**^p < 0.01, ^***^p < 0.001.

To explore whether increased oxidative stress in PTZ-kindled mice is relevant to nNOS signaling, we detected hippocampal reactive oxygen species (ROS) level in nNOS^−/−^ mice as well as their wildtype littermates under normal or PTZ kindling conditions. Our results show that hippocampal ROS production, which was measured by the DHE fluorescence intensity, was remarkably enhanced in the wildtype kindled mice in comparison to wildtype control mice, while the hippocampal DHE fluorescence intensity in nNOS^−/−^ kindled mice was dramatically decreased compared to wildtype kindled mice (Figures 5A,B), suggesting PTZ kindling-induced hippocampal ROS production is dependent upon nNOS activity. For DHE fluorescence intensity, two-way ANOVA revealed a significant main effect of drug treatment [*F*_(1, 16)_ = 43.70, *p* < 0.001] and genotype [*F*_(1, 16)_ = 14.22, *p* = 0.002], as well as drug treatment × genotype interaction [*F*_(1, 16)_ = 12.79, *p* = 0.003]. A Tukey *post-hoc* test revealed that WT kindled mice had significant higher level of DHE intensity than WT ctrl mice (*p* < 0.001), and nNOS^−/−^ kindled mice had significant lower level of DHE intensity than WT kindled mice (*p* < 0.001).

### PTZ kindling activates nNOS-dependent MAPK and PI3K/AKT signaling pathways

The MAPK signaling pathway is involved in modulation of various physiological and pathological events. The correlation of the MAPK signaling pathway with ROS has been investigated in many studies (Ramos-Nino et al., 2002; Cakir and Ballinger, 2005; Batra et al., 2012; Lee et al., 2016). The MAPK pathway comprises extra-cellular signal-regulated kinases (ERK 1/2), the p38 kinase, and the stress-activated protein kinase or c-Jun N-terminal kinase (SAPK/JNK) (Seger and Krebs, 1995). To determine whether MAPK signaling pathway is activated in the hippocampus of PTZ-kindled mice and whether this signaling pathway is dependent on nNOS activation, we detected ERK and p38 and their phosphorylation in nNOS^−/−^ mice and their wildtype littermates under normal and PTZ kindling conditions by western blot. Our results revealed that phosphorylation of p38 and ERK in the hippocampus of wildtype kindled mice was significantly increased compared to wildtype control mice. Moreover, the phosphorylation of p38 and ERK in the hippocampus of nNOS^−/−^ kindled mice was significantly decreased compared to that of wild type kindled mice (Figures 5C–E). For ERK phosphorylation, two-way ANOVA revealed a significant main effect of PTZ treatment [*F*_(1, 16)_ = 15.46, *p* = 0.001], genotype [*F*_(1, 16)_ = 9.6, *p* < 0.007], as well as PTZ treatment × genotype interaction [*F*_(1, 16)_ = 9.89, *p* = 0.006]. A Tukey *post-hoc* test revealed that WT kindled mice had higher ERK phosphorylation level than WT ctrl mice (*p* < 0.001), and nNOS^−/−^ kindled mice had significant lower ERK phosphorylation level than WT kindled mice (*p* < 0.001). For p-38 phosphorylation, two-way ANOVA revealed a significant main effect of PTZ treatment [*F*_(1, 16)_ = 50.59, *p* < 0.001], genotype [*F*_(1, 16)_ = 31.06, *p* < 0.001], as well as PTZ treatment × genotype interaction [*F*_(1, 16)_ = 34.93, *p* < 0.001]. A Tukey *post-hoc* test revealed that WT kindled mice had higher p38 phosphorylation level than WT ctrl mice (*p* < 0.001), and nNOS^−/−^ kindled mice had significant lower p38 phosphorylation level than WT kindled mice (*p* = 0.002). These data suggests that PTZ kindling activates nNOS dependent MAPK signaling pathway.

PI3K/AKT signaling pathway is another important signaling pathway which is involved in regulating redox status (Uranga et al., 2013; Hambright et al., 2015). To determine whether PI3K/AKT signaling pathway is activated in the hippocampus of PTZ-kindled mice and whether this signaling pathway is dependent on nNOS activation, we detected PI3K, AKT and phosphorylation of AKT in nNOS^−/−^ mice and their wildtype littermates under normal and PTZ kindling conditions by western blot. Our results showed that PI3K level and phosphorylation of AKT in the hippocampus of wildtype kindled mice was significantly increased compared to wildtype control mice. Moreover, the PI3K level and phosphorylation of AKT in the hippocampus of nNOS^−/−^ kindled mice was significantly decreased compared to that of wild type kindled mice (Figures 5F–H). For PI3K level, two-way ANOVA revealed a significant main effect of PTZ treatment [*F*_(1, 16)_ = 14.63, *p* = 0.001], genotype [*F*_(1, 16)_ = 10.55, *p* < 0.005], as well as PTZ treatment × genotype interaction [*F*_(1, 16)_ = 5.49, *p* = 0.032]. A Tukey *post-hoc* test revealed that WT kindled mice had higher PI3K level than WT ctrl mice (*p* < 0.001), and nNOS^−/−^ kindled mice had significant lower PI3K level than WT kindled mice (*p* < 0.001). For AKT phosphorylation level, two-way ANOVA revealed a significant main effect of PTZ treatment [*F*_(1, 16)_ = 16.29, *p* < 0.001], genotype [*F*_(1, 16)_ = 10.84, *p* = 0.005], as well as PTZ treatment × genotype interaction [*F*_(1, 16)_ = 15.40, *p* = 0.001]. A Tukey *post-hoc* test revealed that WT kindled mice had higher AKT phosphorylation level than WT ctrl mice (*p* < 0.001), and nNOS^−/−^ kindled mice had significant lower AKT phosphorylation level than WT kindled mice (*p* < 0.001). These data suggests that PTZ kindling activates nNOS dependent PI3K/AKT signaling pathway. Taken together, these results suggest that PTZ kindling activates both MAPK and PI3K/AKT signaling pathways and the activation of these signaling pathways are dependent on nNOS activation.

## Discussion

Cognitive dysfunction and depressive like behavior have been reported as main neurobehavioral comorbidities of chronic epilepsy, which significantly impact the outcomes and affects the life quality of epilepsy patients. Cognitive impairment in is evident in children with epilepsy. It is reported that children with generalized nonabsence seizures were at increased risk for learning abilities (Zalachoras et al., 2016). Furthermore, Children who have pharmacoresistant seizures appear to have lower IQ scores than children with well controlled seizures (Guo and Commons, 2017). Adults with chronic epilepsy are also reported to have cognitive impairment (Hutton et al., 2017; Shrestha et al., 2017). Depression is regarded as the most common comorbid condition of epilepsy, with prevalence in the range of 25–55% in epilepsy patients. Moreover, the incidence of depression is remarkably higher in epilepsy patients than that in the normal people (Cramer et al., 2003). PTZ Kindling is a well-established chronic epilepsy model that has been extensively studied to understand the pathological mechanisms of epilepsy. Here in this study, we found that PTZ kindling triggered cognition impairment and depressive like behavior, which is in agreement with previous reports (Russo et al., 2013; Loughman et al., 2016; Tai et al., 2016). Although epilepsy is known to be relevant to a high incidence of cognition deficits and depressive-like behavior, the responsible underlying mechanisms remains elusive. NO has been recognized as a neuronal messenger, which is involved in regulation the balance of neurotransmission (West et al., 2002; Garthwaite, 2008; Raju et al., 2015). It is suggested that alterations of NO signaling may contribute to the pathophysiology of cognition deficits (Walton et al., 2013; Funk and Kwan, 2014) and depression (Zhou et al., 2007; Gigliucci et al., 2014). A previous study demonstrated that nNOS accounts for approximately 90% of the overall NO production in the brain (Hara et al., 1996), suggesting that nNOS is mainly responsible for the NO signaling-mediated pathophysiological process in the brain. Our previous study and others' reported that PTZ-kindling enhanced hippocampal nNOS expression and enzymatic activity (Itoh et al., 2004; Zhu et al., 2016a). However, this PTZ kindling-induced increase of nNOS signaling is abolished in nNOS knockout mice. nNOS knockout mice are viable and show normal behavior, although they exhibit enlarged stomachs and dysfunction in gastrointestinal motility (Huang, 1999). When nNOS knockout mice are subjected to focal ischemia, they have smaller infarcts, suggesting a protective role of nNOS against neurotoxicity (Huang, 1999). Interestingly, genetic deletion of nNOS did not affect the PTZ kindling progress, which is consistent with a previous report (Itoh and Watanabe, 2009). To define a primary role of nNOS on PTZ kindling-induced psychiatric comorbidities, we measured the cognition function and depressive-like behavior in nNOS deficient mice and their wildtype littermates under normal and PTZ kindling conditions. We demonstrated that PTZ kindling-induced cognition deficit and depressive-like behavior is dependent on nNOS activity. These results suggest that nNOS plays a crucial role in PTZ kindling-induced psychiatric comorbidities.

Redox homeostasis is essential for maintain normal function of brain. Excessive production of ROS, a hallmark of redox homeostasis impairment in the brain, appears to be involved in the pathogenesis of epilepsy (Rowley et al., 2015; Williams et al., 2015). In agreement with previous studies, here we show that hippocampal ROS level are significantly increased in PTZ-kindled mice, however, depletion of nNOS suppressed PTZ kindling-induced ROS production, indicating PTZ kindling-induced ROS production is dependent on nNOS activity. Mounting evidence suggests that increased oxidative stress in the brain was usually accompanied with cognition deficit and depressive-like behavior. (de Morais et al., 2014; Pearson et al., 2015; Taiwe et al., 2015). Both MAPK and PI3K/AKT signaling pathways have been reported to respond to ROS stimulation in the central nervous system, thereby activating certain cellular events which contribute to pathological processes (Shah et al., 2014; Brobey et al., 2015). A recent study reported that ROS-mediated MAPK signaling pathway activation plays a pivotal role in cognition deficits in Alzheimer's disease (Arora et al., 2015). Our data show that both MAPK and PI3K/AKT signaling pathways have been activated in PTZ-kindled mice, and both signaling pathways activation are dependent on nNOS activity, suggesting nNOS may activate MAPK and PI3K/AKT signaling pathways through ROS production to trigger PTZ kindling-induced cognition deficit and depressive-like behavior.

In summary, here we have used a PTZ kindling epilepsy model, supported by a genetic nNOS deficient mice, to demonstrate nNOS as a critical signaling in PTZ kindling -induced comorbidities including cognitive impairment and depressive-like behavior. Our understanding of the role of nNOS signaling in PTZ kindling-induced cognition deficit and depressive-like behavior may provide insight into the molecular mechanism for psychiatric comorbidities in chronic epilepsy patients.

## Author contributions

XZ, JC, and HY designed research; XZ, JD, BH, RH, AZ, ZX, and HC performed research; XZ analyzed data and wrote the paper.

### Conflict of interest statement

The authors declare that the research was conducted in the absence of any commercial or financial relationships that could be construed as a potential conflict of interest.
